# Idebenone improves post-thaw motility and mitochondrial function of buck sperm

**DOI:** 10.5713/ab.260339

**Published:** 2026-09-01

**Authors:** Min Jung Kim, Seungjun Lee, Ahmad Yar Qamar, Jaeuk Cho, Minwoo Sin, Chanjin Woo, Inhwan You, Muhammad Bilal, Yunju Ha, Seeun Kim, Sang-Hee Lee, Jongki Cho

**Affiliations:** 1 College of Veterinary Medicine and Research Institute for Veterinary Science, Seoul National University, Seoul, Korea; 2 Mjbiogen Corporation, Namyangju, Korea; 3 College of Veterinary and Animal Sciences, Jhang Sub-campus of University of Veterinary and Animal Sciences, Lahore, Pakistan; 4 College of Animal Life Science, Kangwon National University, Chuncheon, Korea; 5 Farm Animal Clinical Training and Research Center, Institutes of Green-Bio Science and Technology, Seoul National University, Pyeongchang, Korea

**Keywords:** Antioxidant, Buck Sperm, Cryoprocessing, Idebenone, Mitochondrial Activity

## Abstract

**Objective:**

This study was performed to evaluate the effects of idebenone (ID) supplemented freezing extender on the post-thaw quality of buck sperm, with particular focus on sperm kinematic parameters, viability, acrosome integrity, mitochondrial function, and antioxidant-related gene expression.

**Methods:**

Semen samples collected from five sexually active bucks were diluted in freezing extender supplemented with different concentrations of ID (0, 1, 5, and 10 μM). Post-thaw sperm kinematic parameters were analyzed using computer-assisted sperm analysis, whereas sperm viability, acrosome integrity, mitochondrial activity, and mitochondrial membrane integrity were assessed by flow cytometry using SYBR-14/DRAQ7, FITC-PNA/DRAQ7, MitoTracker Green/DRAQ7, and Rhodamine 123/DRAQ7 staining, respectively. The relative mRNA expression levels of glutathione peroxidase (GPX) and superoxide dismutase (SOD) were measured by real-time polymerase chain reaction using β-actin as a reference gene.

**Results:**

The ID5 group showed improved total and progressive motility compared with the control group among the tested concentrations. Based on these findings, ID5 was selected as optimal concentration for cryoprocessing and subsequently used for further functional evaluation. Sperm viability exhibited no significant differences between the control and ID5 groups. In contrast, sperm acrosomal integrity and mitochondrial activity were significantly improved in the ID5 group compared with the control. Mitochondrial membrane integrity was not significantly different between the control and ID5 groups. In gene expression analysis, GPX levels were significantly reduced in the ID5 group, whereas SOD2 expression was not significantly changed.

**Conclusion:**

These results suggest that freezing extender supplemented with 5 μM, as an optimum level of ID can improve the post-thaw quality of buck sperm. The beneficial effects of ID5 appear to be associated with improved motility, acrosomal status, and mitochondrial function, indicating its potential as a useful additive for buck sperm cryoprocessing.

## INTRODUCTION

Cryoprocessing of buck semen is considered a prerequisite for artificial insemination, preservation of elite genetics, and propagation of breeding traits with high value. However, the cryoprocessing causes substantial structural and functional damage to sperm, resulting in reduced motility, plasma membrane integrity, acrosome status, and fertility potential [1,2]. During cryoprocessing, sperm are exposed to cold shock, osmotic stress, and oxidative stress (OS) resulting in lipid peroxidation, mitochondrial dysfunction, and DNA damage [3–6]. Buck sperm are particularly vulnerable to cryo-injury because of seminal plasma lipase that can interact with different components of freezing extender, such as egg yolk lipids, producing toxic substances that destabilize the plasma membrane of sperm during cryoprocessing [2]. Moreover, egg yolk used in the freezing extender reacts with glycoprotein-60 (BUSgp60) found in the secretions of bulbourethral gland of buck having triacylglycerol hydrolase activity that reduces sperm motility by affecting structural integrity of sperm plasma membrane [7] while phospholipase A_2_ regulates the hydrolysis of phosphatidylcholine found in the egg yolk into fatty acids and lysophophatidylcholine that has detergent effect on biological membranes. Both triacylglycerol hydrolase and phospholipase A_2_ trigger cryocapacitation, reduced sperm motility and viability, and acrosomal damage [8]. To overcome these issues, washing of buck sperm immediately after semen collection was included in the cryoprocessing protocol [9]. However, the washing process can result in the depletion of beneficial seminal plasma constituents, including protective proteins and antioxidant enzymes that play a crucial role in maintaining sperm integrity [10].

As compared to other cells, sperm lack the cytoplasmic content that usually contains an antioxidant defense system against reactive oxygen species (ROS). Moreover, the higher content of polyunsaturated fatty acids (PUFAs) in the plasma membrane makes buck sperm highly susceptible to oxidative damage, resulting in decreased post-thaw viability and fertilizing capacity [4,11]. Therefore, providing antioxidant defense during cryoprocessing is considered a key strategy to enhance the post-thaw survival and functional competence of buck sperm [5,12].

During sperm cryoprocessing, supplementation of freezing extender with antioxidants has been widely studied as an effective approach to reduce OS [12–15]. Previous studies have shown that supplementing freezing extender with antioxidants, including glutathione, plant-derived compounds, and mitochondrial-targeted antioxidants, can improve post-thaw sperm motility, membrane integrity, and fertilizing ability by decreasing ROS levels and enhancing antioxidant enzyme activity [3,16–18]. Recent studies have also evaluated other extender additives, including antioxidant nanomaterials, nanoemulsions, growth factor-enriched cryo-agents, and combined cryoprotectants, to improve post-thaw sperm quality in different mammalian species [19–23]. Maintenance of mitochondrial function is particularly important for sperm survival because ATP production and mitochondrial membrane potential directly regulate sperm motility and fertilizing ability, and both are highly sensitive to oxidative damage during post-thaw [4,18]. Idebenone (ID) is a synthetic analog of coenzyme Q10 with strong antioxidant activity and the ability to support mitochondrial electron transport, thereby reducing oxidative damage to cellular lipids, proteins, and DNA [24]. Due to its protective nature, ID may help to preserve sperm function through improved mitochondrial integrity during sperm cryoprocessing in ram and boar [25,26]. However, its effects on the cryoprocessing of buck sperm have not yet been clearly investigated.

Therefore, the present study aimed to evaluate the effects of ID-supplemented freezing extender on the post-thaw quality-related parameters of buck sperm. Post-thaw sperm characteristics were assessed using computer-assisted sperm analysis (CASA) to determine motility-related parameters, flow cytometry to evaluate plasma membrane integrity, acrosome status, mitochondrial activity, and oxidative damage, and real-time polymerase chain reaction (PCR) to analyze the expression of antioxidant-related genes. This study was designed to determine whether ID can improve the cryosurvival of buck sperm by enhancing antioxidant capacity and maintaining cellular function during cryoprocessing.

## MATERIALS AND METHODS

### Chemical use

All chemicals were purchased from Sigma-Aldrich, unless otherwise indicated.

### Semen collection and processing

Semen samples were collected from five healthy, sexually active bucks (aged 2−5 years, average body weight 30−45 kg) maintained at the experiment goat farm of Kangwon National University (Republic of Korea). Bucks are seasonal breeders, and semen was collected once a week from December to January. All five bucks were clinically healthy and had no history of reproductive disorders. Before semen collection, the bucks were restrained in a holding frame, and faeces were manually removed from the rectum. The hairs around the preputial area were clipped and disinfected prior to the procedure. Semen was collected using an electroejaculation method with an ejaculator (Lane Manufacturing) by inserting a rectal probe and applying electrical stimulation.

Semen samples with live sperm (>80%), progressive motility (>70%), and sperm concentration (>3×10^9^ sperm/mL) were pooled together and diluted fourfold with a first buffer consisting of Tris (hydroxymethyl) aminomethane and citric acid dissolved in distilled water (pH 7.0). The diluted semen was centrifuged at 700×g for 15 min at 24°C to remove seminal plasma. After centrifugation, the supernatant was discarded, and the sperm pellet was resuspended with an equal volume of freezing extender consisted of triple-distilled water, Triladyl (Minitube), and egg yolk at a ratio of 60:20:20 (v/v/v) to achieve a final concentration of 2×10^8^ sperm/mL.

For cryopreservation, diluted semen was cooled gradually from 38°C to 24°C over 1 h, followed by further cooling to 4°C over 2 h to achieve equilibration. After equilibration, cooled semen was filled into 0.25 mL French straws sealed with polyvinyl alcohol powder. Following sealing, the semen straws were subject to freezing by placing the straws 4 cm above liquid nitrogen for 15 min and then plunged into liquid nitrogen for long-term storage until analysis. For post-thaw analysis, frozen semen straws were thawed in a 38°C water bath for 45 s. The thawed semen was diluted with Modena B extender to adjust the final concentration to 2×10^7^ spermatozoa/mL, and the samples were immediately used for subsequent analysis.

### Experimental design

To evaluate the effect of ID supplementation on cryoprocessing of buck sperm, ID was added to the freezing extender at final concentrations of 0 μM (control), 1 μM (ID1), 5 μM (ID5), and 10 μM (ID10). After dilution with the freezing extender, semen samples were cryopreserved as described above. Semen was collected on five different days. Each collection day was considered one independent biological replicate, and CASA analysis was performed using all five collections (n = 5).

After thawing, sperm kinematic parameters were evaluated using CASA (Sperm Class Analyzer System; Microptic) to compare all experimental groups. Based on the CASA results, the group showing the most favorable sperm motility was selected for further analysis. Flow cytometry and quantitative real-time PCR analysis were then performed using only the control group and the optimal concentration group to evaluate sperm viability, acrosome integrity, mitochondrial function, mitochondrial membrane potential, and antioxidant-related gene expression.

### Sperm kinematic parameters analysis

Sperm concentration and kinematic parameters were evaluated using a CASA [27]. The system was connected to a phase-contrast microscope equipped with a heating stage maintained at 37°C. All settings of the CASA system were adjusted according to the manufacturer’s recommendations. For analysis, thawed semen samples were loaded into a pre-warmed chamber slide (Leja) and examined under a phase-contrast microscope. At least five different microscopic fields were analyzed for each sample, and sperm motility parameters were evaluated automatically using the CASA system. Various parameters such as total motility, progressive motility, curvilinear velocity (VCL), straight-line velocity (VSL), average path velocity (VAP), linearity (LIN), straightness (STR), wobble (WOB), amplitude of lateral head displacement (ALH), and beat cross frequency (BCF) were analyzed.

### Flow cytometry analysis

Flow cytometry analysis was performed to evaluate sperm viability, acrosome integrity, mitochondrial activity, and mitochondrial membrane potential (ΔΨm) as previously explained. Post–thawed semen samples were diluted to 1×10^7^ sperm/mL in modified Modena B extender before staining. Sperm viability was assessed using SYBR-14 (Invitrogen) and DRAQ7 (Biostatus, UK). Diluted semen samples were incubated with SYBR-14 and DRAQ7 for 15 min at 37°C. Live sperm were stained green, whereas membrane-damaged sperm were stained red.

Acrosome integrity was evaluated using fluorescein isothiocyanate-conjugated peanut agglutinin (FITC-PNA) combined with DRAQ7. Samples were incubated with FITC-PNA and DRAQ7 for 15 min at 37°C and then analyzed by flow cytometry. Acrosome intact sperm exhibited negative FITC-PNA staining, whereas acrosome reacted sperm was identified by a significant shift in green fluorescence compared to acrosomes that did not react. Mitochondrial activity was analyzed using MitoTracker Green (Invitrogen) with DRAQ7. Samples were incubated with MitoTracker Green for 5 min at 37°C, followed by DRAQ7 for 10 min at 37°C before analysis. Mitochondrial membrane integrity was evaluated using Rhodamine 123 (R123) with DRAQ7 staining to confirm mitochondrial function. Samples were evaluated with R123 for 15 min at 37°C, and DRAQ7 for 10 min at 37°C in the dark. All stained samples were analyzed using a flow cytometer (Thermo Fisher Scientific). A total of 10,000 gated events were recorded for each sample. Fluorescence signals were detected using forward scatter (FSC), side scatter (SSC), green fluorescence (FL1), and red fluorescence (FL2) channels.

For quantitative analysis, debris and non-sperm events were first excluded based on FSC and SSC properties. Sperm viability was calculated as the percentage of SYBR-14-positive sperm, acrosome integrity as the percentage of FITC-PNA-negative sperm, mitochondrial activity as the percentage of MitoTracker Green-positive sperm, and mitochondrial membrane integrity as the percentage of R123-positive sperm. The percentages shown in the representative dot plots indicate the distribution of events in one representative sample, whereas the bar graphs were generated from the mean±standard error of the mean (SEM) of five independent biological replicates (n = 5) using the same gating strategy.

### Real-time polymerase chain reaction analysis

Total RNA was extracted from post-thaw sperm both from ID-5 and control groups using TRIzol reagent (Thermo Fisher Scientific) according to the manufacturer’s instructions [27]. The concentration of extracted RNA was measured using a Qubit 4 Fluorometer (Thermo Fisher Scientific). Complementary DNA (cDNA) was synthesized using Maxime RT PreMix Kit with Oligo (dT)15 Primer (iNtRON Biotechnology) following the manufacturer’s protocol. Quantitative real-time PCR was performed using PowerUp SYBR Green Master Mix (Applied Biosystems; Thermo Fisher Scientific) on a QuantStudio 1 Real-Time PCR System (Applied Biosystems). Each reaction mixture contained forward primer, reverse primer, SYBR Green Master Mix, nuclease-free water, and cDNA in a total volume of 20 μL. The amplification conditions were as follows: initial denaturation at 95°C, followed by 40 cycles of denaturation at 95°C, annealing at 60°C, and extension at 72°C. Primer sequences used in this study are listed in Table 1. The expression levels of target genes were normalized to the housekeeping gene β-actin (ACTB). Relative gene expression levels were calculated using the ΔCt method, and statistical analysis was performed based on ΔCt values.

### Statistical analysis

Data were analyzed using GraphPad Prism version 8 (GraphPad Software). All the values are presented as mean±SEM. Data related to the total motility and kinematic parameters used to determine the optimum level of ID were analyzed using one-way analysis of variance (ANOVA) followed by Tukey’s multiple comparison test. Flow cytometry and real-time PCR data were analyzed using a paired t-test. Differences were considered statistically significant at p<0.05.

## RESULTS

### Effect of idebenone on sperm kinematic parameters after cryopreservation

The effects of different concentrations of ID on sperm kinematic parameters after cryopreservation are shown in Table 2. Total motility was significantly higher in the ID5 group (55.5±1.3%) as compared with the control (46.3±2.1%) and ID1 groups (45.0±3.7%), respectively. The ID10 group (49.4±0.9%) did not show a significant difference compared with the other groups. Progressive motility also differed among groups. The ID5 group showed significantly higher progressive motility (24.3±2.4%) than the control group (18.4±3.2%). Whereas, the ID1 (22.0±3.1%) and ID10 (22.0± 1.5%) groups did not show significant differences with the control and ID5 groups. For other kinematic parameters, including VCL, VAP, VSL, STR, LIN, WOB, ALH, and BCF, no significant differences were observed among the experimental groups.

### Effect of idebenone on post-thaw sperm viability and acrosome integrity

Flow cytometric analysis was performed to evaluate sperm viability and acrosome integrity in post-thaw buck sperm supplemented with ID (Figure 1). Sperm viability assessed by SYBR-14/DRAQ7 staining was not significantly different between the control and ID5 groups (46.8±2.7% vs 55.5±7.7%, p = 0.36, Figure 1A). In contrast, acrosome integrity assessed by FITC-PNA/DRAQ7 staining was significantly increased in the ID5 group (57.1±8.9%) compared with the control group (52.2±8.9%, p = 0.006, Figure 1B). Results demonstrated that ID supplementation during cryoprocessing improved sperm functional integrity, particularly by enhancing the percentage of acrosome intact sperm, while sperm viability showed only a tendency to increase without statistical significance.

### Effect of idebenone on post-thaw sperm mitochondrial activity and membrane integrity

Flow cytometric analysis was performed to evaluate mitochondrial activity and mitochondrial membrane integrity in post-thaw buck sperm following ID supplementation (Figure 2). Mitochondrial activity assessed by MitoTracker Green/DRAQ7 staining was significantly improved in the ID5 group (53.5±5.1%) compared to the control (41.1±0.9%, p = 0.042, Figure 2A). In contrast, mitochondrial membrane integrity evaluated using R123/DRAQ7 staining was not significantly different between the control group (39.1±4.5%) and the ID5 group (43.6±2.9%, p = 0.32, Figure 2B).

### Effect of idebenone on antioxidant-related gene expression

The expression levels of antioxidant-related genes in post-thawed buck sperm were analyzed by real-time PCR (Figure 3). GPX showed a distinct expression pattern between the control and ID5 groups. In the relative expression analysis, GPX expression was markedly lower in the ID5 group than in the control group, corresponding to an approximately five-fold decrease. This result suggests that ID supplementation during cryoprocessing was associated with a noticeable change in the expression of a glutathione-related antioxidant gene. However, since GPX expression was evaluated only at the transcriptome level, these results should be interpreted cautiously as treatment-related changes in redox-related gene expression rather than as direct mechanistic evidence for the reduction of OS. SOD2 showed a different pattern from GPX. The ΔCt values of SOD2 were not significantly different between the control and ID5 groups, suggesting that ID supplementation did not clearly alter SOD2 expression after cryoprocessing. In the relative expression analysis, SOD2 expression was slightly higher in the ID5 group than in the control group, but this difference was not statistically significant. Taken together, these results indicate that the effect of ID supplementation on antioxidant-related gene expression was mainly observed in GPX, whereas SOD2 expression remained relatively unchanged in post-thawed buck sperm.

## DISCUSSION

Improving the resistance of buck sperm to freezing and thawing was a major reason for optimizing the extender composition in this study. During cryoprocessing, spermatozoa undergo cold shock, osmotic stress, and membrane remodeling, and these changes can increase ROS production. In ruminant sperm, ROS-mediated damage is especially relevant because the sperm membrane contains abundant PUFAs, while the cytoplasmic antioxidant reserve is limited. When this balance is disrupted, lipid peroxidation can occur, followed by loss of membrane integrity, reduced mitochondrial activity, and decreased post-thaw motility [16,20,28]. Recent LC-MS metabolomic analysis of goat semen further showed that cryopreservation is associated with marked metabolic alterations and potential biomarkers of cryo-injury, supporting the view that freezing and thawing affect multiple cellular pathways [29]. For this reason, we considered antioxidant supplementation as a practical strategy to reduce cryo-induced injury in buck sperm. Exogenous antioxidants may protect spermatozoa by limiting lipid peroxidation, preserving mitochondrial function, and supporting the remaining endogenous antioxidant defense after thawing [18,30,31]. However, antioxidant treatment cannot be interpreted simply as “more is better.” A low concentration may be insufficient to counteract freezing-induced oxidative damage, whereas an excessive concentration may disturb the physiological redox balance required for sperm function. Therefore, the concentration of the antioxidant additive was treated as an important experimental variable in the present study [16,28]. Therefore, identifying an optimal antioxidant type and concentration is critical for improving the efficiency of buck sperm cryoprocessing.

In the present study, ID supplementation influenced post-thaw sperm motility, and the improvement observed at the optimal concentration (Figure 1) suggests that antioxidant supplementation can enhance sperm resistance to cryodamage. Similar results have been reported in previous studies in which the addition of antioxidants to freezing extenders improved total and progressive motility after thawing by reducing oxidative damage and preserving cellular function during cryoprocessing [4,30]. Cryoprocessing is known to impair sperm motility through OS-induced lipid peroxidation of the plasma membrane and mitochondrial dysfunction. These changes reduce ATP production required for flagellar movement [16,28]. Therefore, the inclusion of antioxidants in the freezing extender can help maintain motility by stabilizing membrane lipids and protecting mitochondrial activity during the cryoprocessing. The results of the present study demonstrated a dose-dependent effect of ID supplementation, since the moderate level of ID was found to be effective in improving quality-related parameters of buck sperm, whereas the higher level failed to trigger further improvement. Similar findings have been reported by several other studies on sperm cryoprocessing, in which optimum levels of antioxidants improved sperm quality, whereas excessive levels proved detrimental due to redox imbalance or metabolic disturbance [4,16]. Moreover, freezing extender supplemented with higher levels of antioxidants may disrupt the physiological level of ROS required for normal sperm function, resulting in reduced sperm motility. These findings suggest that determining the optimal concentration of antioxidant additives is critical for improving the efficiency of cryoprocessing of buck sperm.

In addition to motility, the integrity of the plasma membrane and acrosome is considered a key indicator of sperm functionality after cryopreservation [32]. Moreover, it has been reported that the combined assessment of membrane and acrosome integrity is more closely related to fertility than motility alone, emphasizing the importance of multi-parameter analysis in cryopreservation studies [33]. Cryoprocessing can induce structural damage to the sperm plasma membrane, resulting in increased permeability, loss of acrosomal integrity, and reduced fertilizing capacity [16,28]. Acrosomal contents play an imperative role in the penetration of the zona pellucida around oocytes; even a motile sperm with a damaged acrosome fails to fertilize the oocyte [33]. Previous studies have reported similar findings, showing that antioxidant-supplemented freezing extenders increased the proportion of post-thaw sperm with an intact plasma membrane by reducing lipid peroxidation and oxidative injury to membrane phospholipids [18,30]. The ID-supplemented group showed a higher percentage of acrosome-intact sperm after thawing. We interpreted this result as evidence that ID reduced cryo-induced damage to membrane-associated sperm structures. This point is important because the acrosome is not only a morphological marker. It is a membrane-bound structure that must remain intact until the sperm reaches the oocyte. When the acrosomal membrane is damaged during freezing and thawing, spermatozoa may fail to undergo a normal acrosome reaction, even if some motility is still preserved. Therefore, the increase in acrosome-intact sperm suggests that ID affected more than post-thaw movement; it also helped maintain a structure directly linked to fertilizing ability. We assessed this change using fluorescent staining and flow cytometric analysis. This approach allowed us to classify individual sperm cells according to plasma membrane status and acrosome integrity under the same analytical conditions. Compared with observation based only on microscopic fields, flow cytometry provided a more quantitative assessment of post-thaw sperm quality and reduced the possibility of observer-dependent variation [34,35].

In this study, we considered mitochondrial preservation as an important basis for explaining the improvement in post-thaw sperm quality. In spermatozoa, mitochondria are concentrated in the midpiece, where they support the energy demand required for flagellar movement. Therefore, when mitochondrial activity declines after freezing and thawing, the effect can appear directly as reduced motility and lower sperm survival [36,37]. Cryoprocessing can disturb mitochondrial function through several types of stress, including cold shock, osmotic changes, and membrane phase transitions. These stresses can disrupt mitochondrial membrane potential (ΔΨm), increase ROS production, and promote oxidative injury [17]. We regarded this pathway as particularly relevant to mammalian sperm because their membranes contain abundant PUFAs. Although these lipids contribute to membrane flexibility, they are also highly vulnerable to ROS-induced lipid peroxidation during freezing and thawing [33]. Based on this mechanism, mitochondrial damage was not treated as a secondary event in the present study. Instead, we interpreted it as one of the main processes linking cryo-induced OS with reduced motility, viability, and fertilizing ability. Reduction of mitochondrial activity can limit ATP production and may also accelerate apoptotic-like changes, which together contribute to the decline in post-thaw sperm function [18,30].

ID is a synthetic analogue of coenzyme Q10, its quinone structure allows it to participate in mitochondrial electron transfer, and previous reports have linked ID treatment with reduced oxidative injury under stress conditions. From a formulation perspective, ID is also easier to apply than CoQ10, which is limited by poor aqueous solubility. This point is important in semen cryopreservation because an antioxidant additive should be dispersed as evenly as possible in the freezing medium. In the present study, however, we used ID only as an extender supplement. We did not examine its intracellular localization, mitochondrial accumulation, or direct regulation of local ROS production [24].

The present findings are generally consistent with previous reports in ram and boar sperm, suggesting that the protective effect of ID may be partly conserved across species. However, its optimal concentration and practical application should be optimized according to species and preservation conditions. Taken together, the improvement in mitochondrial activity observed in the present study suggests that ID may have protected sperm from cryo-induced mitochondrial dysfunction, contributing to the overall preservation of post-thaw sperm quality.

To further assess the antioxidant effect of ID during post-thaw procedures, the expression of antioxidant-related genes was evaluated (Figure 3). Sperm possess endogenous antioxidant mechanisms comprising *SOD* and *GPX* enzymes, which play important roles in protecting cellular structures from oxidative damage caused during cryoprocessing [17,28]. In the present study, GPX and SOD2 were evaluated at the mRNA level. GPX mRNA expression was decreased in the ID5 group, whereas SOD2 expression was not significantly changed. In this context, a recent ram semen study showing that GPX supplementation improved post-thaw sperm quality supports the relevance of GPX-related antioxidant responses during sperm cryopreservation [21]. This result suggests that ID supplementation may affect GPX-related antioxidant gene expression during sperm cryopreservation. Previous studies have demonstrated that cryoprocessing increases ROS production beyond the capacity of the intrinsic antioxidant system, resulting in lipid peroxidation, mitochondrial dysfunction, and reduced sperm viability [18,30]. Thus, ID supplementation did not induce a uniform increase in antioxidant-related gene expression. However, the marked decrease in GPX mRNA expression was not accompanied by a similar change in SOD2 expression. This result suggests that ID did not uniformly increase antioxidant-related gene expression under the present experimental conditions.

## CONCLUSION

Taken together, the present results indicate that the use of ID-supplemented freezing extender during cryoprocessing of buck sperm can improve post-thaw sperm quality when applied at an appropriate concentration. The improvement observed in motility, acrosome integrity, and mitochondrial activity suggests that ID contributes to the protection of sperm from cryo-induced oxidative damage. Previous studies have reported that the effectiveness of antioxidant supplementation depends on the concentration used, and excessive levels may disrupt the physiological redox balance required for normal sperm function [16,28]. In agreement with those findings, the moderate concentration showed beneficial effects, whereas the higher concentration did not provide additional improvement. The changes observed in antioxidant-related gene expression further suggest that ID may influence the intracellular oxidative balance during cryopreservation.

## Figures and Tables

**Figure 1:**
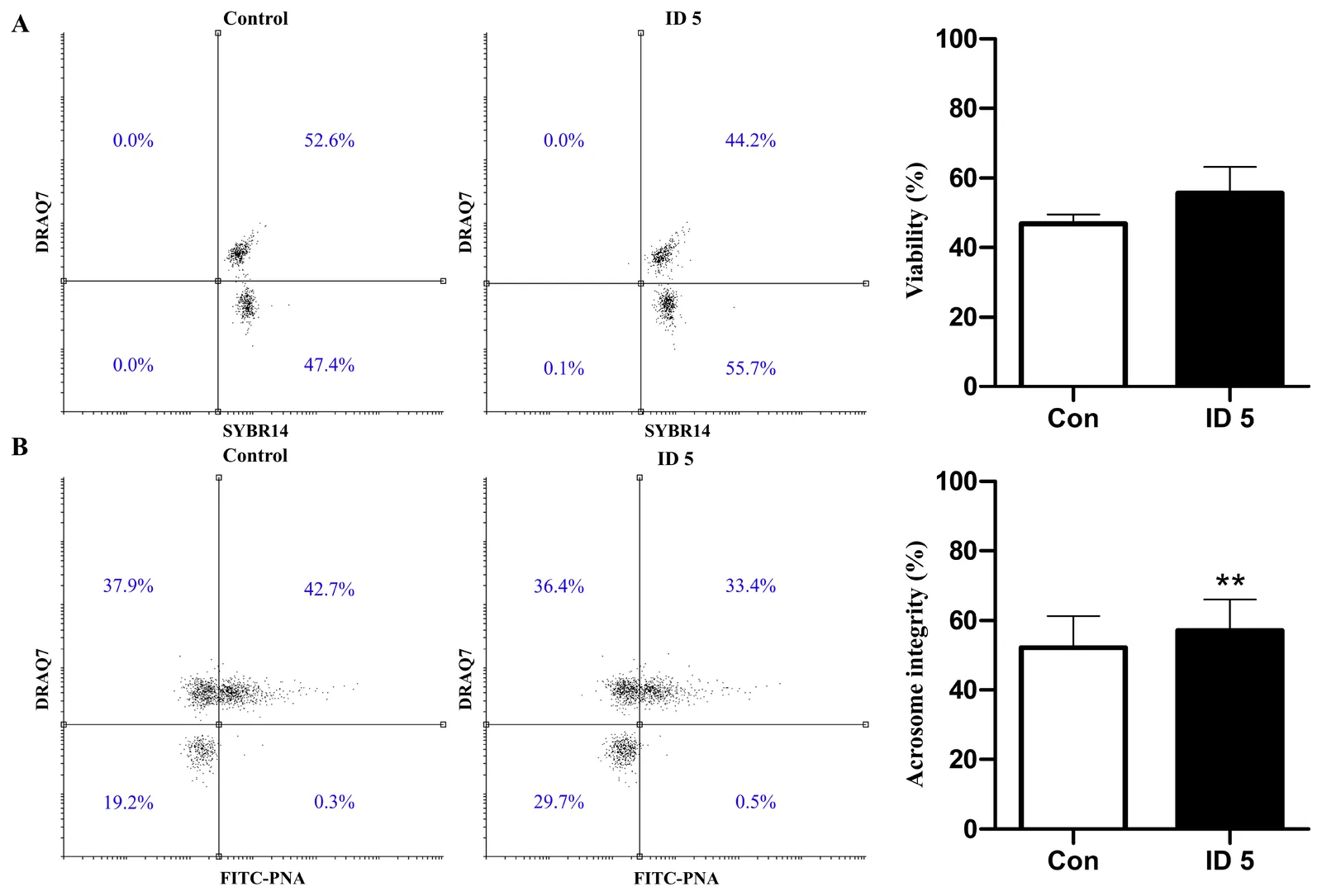
Flow cytometry analysis of frozen–thawed buck sperm viability using SYBR-14/DRAQ7 staining (A) and percentage of intact acrosomes using FITC-PNA/DRAQ7 staining (B) after idebenone (ID) treatment. The dot plots show representative samples, whereas the bar graphs show the mean±SEM from five independent biological replicates (n = 5). ** p<0.01. SEM, standard error of the mean.

**Figure 2:**
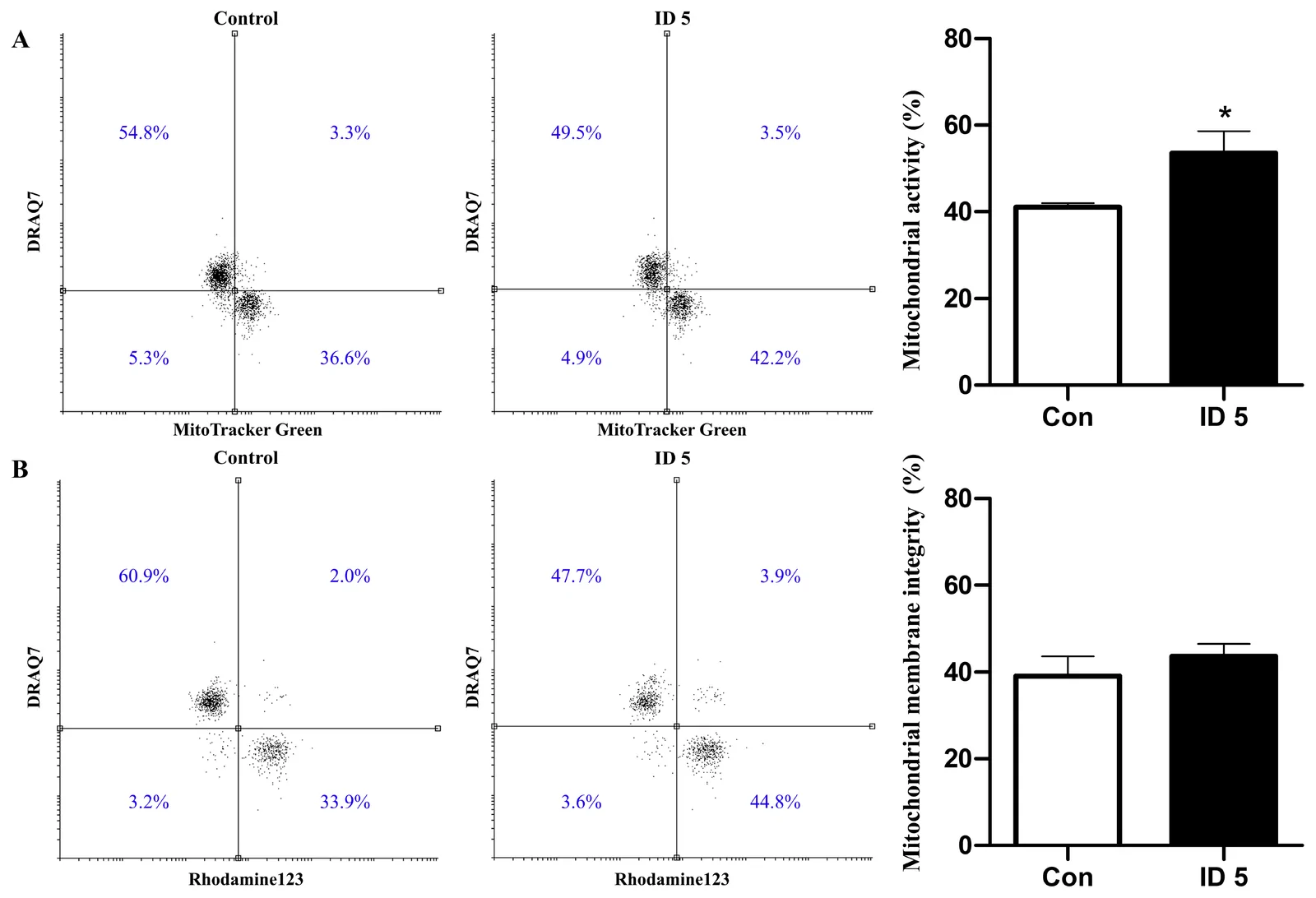
Flow cytometry analysis of frozen–thawed buck sperm mitochondrial activity using MitoTracker Green/DRAQ7 staining (A) and mitochondrial membrane potential using Rhodamine 123/DRAQ7 staining (B) after idebenone (ID) treatment. The dot plots show representative samples, whereas the bar graphs show the mean±SEM from five independent biological replicates (n = 5). * p<0.05. SEM, standard error of the mean.

**Figure 3:**
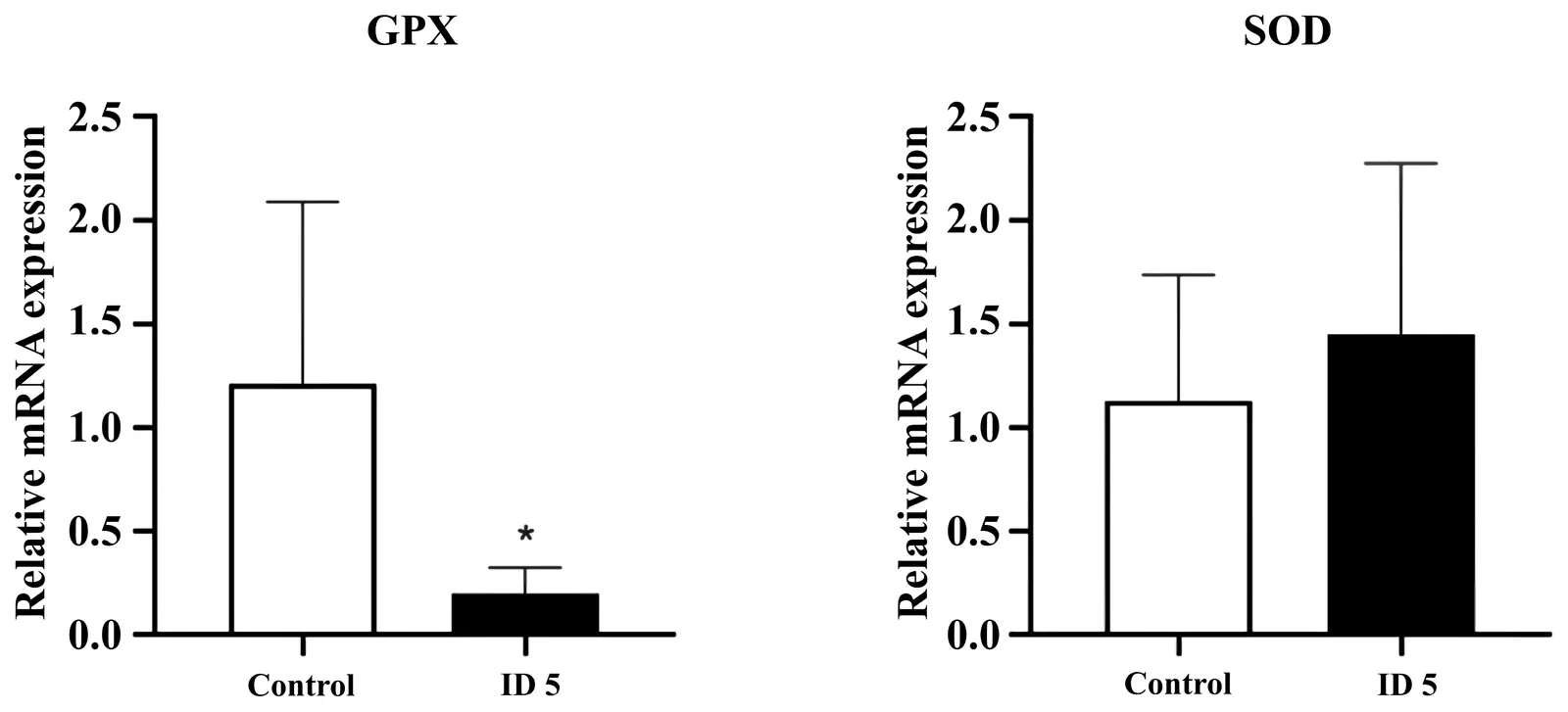
Relative mRNA expression levels of glutathione peroxidase (GPX) and superoxide dismutase (SOD) in frozen–thawed buck sperm after idebenone (ID) treatment. The bar graphs show the mean±SEM from five independent biological replicates (n = 5). * p<0.05. SEM, standard error of the mean.

**Table 1:** Primer sequences used for real-time PCR analysis of buck sperm

Gene	Sequences (5′–3′)		Product size (bp)	Accession No.
*ACTB*	F	gatctggcaccacaccttct	106	NM_001314342.1
	R	catcttctcacggttggcct		
*SOD2*	F	ttgtgaccgaagcagcagaa	99	XM_018053428.1
	R	cttcgtccctcatcctgctc		
*GPX*	F	tgtggtgaagcggtatggtc	145	NM_001285712.2
	R	gaccatcataggcaccaggg		

PCR, polymerase chain reaction.

**Table 2:** Kinematic parameters of frozen–thawed goat sperm in idebenone (ID)-treated groups

Group	Motility (%)	Progressive motility (%)	VCL (%)	VAP (%)	VSL (%)	STR (%)	LIN (%)	WOB (%)	ALH (%)	BCF (%)
Control	46.3±2.1^a^	18.4±3.2^a^	38.6±14.5	17.7±8.9	11.8±7.5	43.6±5.6	16.2±5.3	36.6±4.3	1.2±0.3	4.0±2.9
ID1	45.0±3.7^a^	22.0±3.1^ab^	33.2±16.4	16.3±9.4	11.5±8.1	52±6.3	25±6.7	43.3±6.0	1±0.3	3.6±2.8
ID5	55.5±1.3^b^	24.3±2.4^b^	43.0±8.4	18.8±4.3	10.5±3.2	43.1±3.1	16.2±1.6	37.2±1.2	1.4±0.3	3.3±1.3
ID10	49.4±0.9^ab^	22.0±1.5^ab^	25.2±5.5	11.1±2.8	6±2.3	44±3.5	17.1±3.4	40.6±4.2	0.9±0.2	2.2±1.0

All results show means±SEM.

a,b Different superscripts differ significantly (p<0.05).

VCL, average curvilinear velocity; VAP, average path velocity; VSL, straight-line velocity; STR, straightness (average value of the ratio VSL/VAP); LIN, linearity (average ratio of VSL/VCL); WOB, wobble; ALH, amplitude of lateral head; BCF, beat cross frequency; SEM, standard error of the mean.

## Data Availability

Upon reasonable request, the datasets of this study can be available from the corresponding author.
